# The Valence Band Structure of the [Ni(Salen)] Complex: An Ultraviolet, Soft X-ray and Resonant Photoemission Spectroscopy Study

**DOI:** 10.3390/ijms23116207

**Published:** 2022-06-01

**Authors:** Petr M. Korusenko, Alexandra V. Koroleva, Anatoliy A. Vereshchagin, Danil V. Sivkov, Olga V. Petrova, Oleg V. Levin, Alexander S. Vinogradov

**Affiliations:** 1 Department of Solid State Electronics, St. Petersburg State University, 7/9 Universitetskaya nab., 199034 Saint Petersburg, Russia; d.sivkov@spbu.ru (D.V.S.); teiou@mail.ru (O.V.P.); asvinograd@yahoo.de (A.S.V.); 2 Department of Physics, Omsk State Technical University, 11 Mira prosp., 644050 Omsk, Russia; 3 Resource Center “Physical Methods of Surface Investigation”, St. Petersburg State University, 7/9 Universitetskaya nab., 199034 Saint Petersburg, Russia; st054051@student.spbu.ru; 4 Department of Electrochemistry, Institute of Chemistry, St. Petersburg State University, 7/9 Universitetskaya nab., 199034 Saint Petersburg, Russia; anatoliy_ve@mail.ru (A.A.V.); levin@chem.spbu.ru (O.V.L.); 5 Institute of Physics and Mathematics, Komi Science Centre, Ural Branch of the Russian Academy of Sciences, 167982 Syktyvkar, Russia

**Keywords:** [Ni(Salen)] complex (NiO_2_N_2_C_16_H_14_), NiN_2_O_2_ coordination center, valence band photoemission (VB PE) spectra, soft X-ray and ultraviolet photoemission spectroscopy, X-ray resonant photoemission spectroscopy (ResPES), participator and spectator Auger decay processes

## Abstract

The valence band photoemission (VB PE) spectra of the [Ni(Salen)] molecular complex were measured by ultraviolet, soft X-ray and resonant photoemission (ResPE) using photons with energies ranging from 21.2 eV to 860 eV. It was found that the Ni 3d atomic orbitals’ (AOs) contributions are most significant for molecular orbitals (MOs), which are responsible for the low-energy PE band at a binding energy of 3.8 eV in the VB PE spectra. In turn, the PE bands in the binding energies range of 8–16 eV are due to the photoionization of the MOs of the [Ni(Salen)] complex with dominant contributions from C *2p* AOs. A detailed consideration was made for the ResPE spectra obtained using photons with absorption resonance energies in the Ni 2p_3/2_, N 1s, and O 1s Near-Edge X-ray Absorption Fine Structure (NEXAFS) spectra. A strong increase in the intensity of the PE band *ab* was found when using photons with an energy 854.4 eV in the Ni 2p_3/2_ NEXAFS spectrum. This finding is due to the high probability of the participator-Auger decay of the Ni *2p*_3/2_^−1^*3d*^9^ excitation and confirms the relationship between the PE band *ab* with the Ni 3d-derived MOs.

## 1. Introduction

The [Ni(Salen)] complex (NiO_2_N_2_C_16_H_14_) with the [N_2_O_2_] Schiff base ligand is a promising monomer for the electrochemical synthesis of electrically conductive polymers. This poly-[Ni(Salen)] polymer can be reversibly oxidized and reduced due to the extended conjugated system of π-bonds and the presence of redox centers, which are Ni or/and salen ligand atoms in different charge states. This feature allows it to be applied in various fields such as heterogeneous catalysis, chemical sensors, environmental protection, sustainable energy, and combustion catalysts [1,2,3,4,5]. In particular, E.V. Beletsky et al. in [6] showed that poly-[Ni(Salen)] can be used as a buffer sublayer between the aluminum substrate and the cathode mass in a lithium-ion battery in order to protect against overcharging. Moreover, this polymer finds application as supercapacitor electrodes, due to its high specific redox capacity [7,8]. Another example of the use of poly-[Ni(Salen)] is the creation of an electrochemical sensor based on it for the accurate determination of oxygen, which allows control of the quality and quantity of dissolved oxygen in commercial samples and water [9]. Details of the fundamental and applied studies of [Ni(Salen)] complexes are given in several recently published reviews [10,11,12].

**Figure 1:**
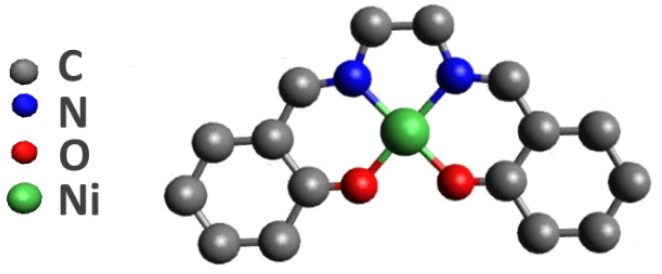
Schematic view of the [Ni(Salen)] complex without hydrogen atoms.

However, the polymerization mechanism of this monomer is not well understood to date. Therefore, detailed knowledge of the atomic-electronic structure of the monomeric [Ni(Salen)] complex is required. The atomic structure of this complex is based on the complexing nickel atom which coordinates two oxygen and two nitrogen atoms of the ligand, forming an almost square-planar [NiO_2_N_2_] coordination center (Figure 1) [13]. An important feature of the [Ni(Salen)] complex is that it is diamagnetic with the total spin S = 0 in the ground state, due to the fact that all valence molecular orbitals (MOs) are completely filled [14,15]. This significantly simplifies acquiring detailed information about the electronic structure of this complex. Various techniques, such as optical, near infrared, and infrared absorption spectroscopy; near edge X-ray absorption fine structure (NEXAFS); and X-ray photoelectron spectroscopy (XPS) and their combinations thereof are used to obtain this information [15,16,17,18].

In reference [14], the characterization of the atomic and electronic structure of the [Ni(Salen)] complex was performed using NEXAFS and XPS, as well as density functional theory (DFT) calculations. It was found that the highest occupied MO (HOMO) in the valence band (VB) is mainly localized on the phenyl rings of the salen ligand at a binding energy (BE) of 2.9 eV. It was also suggested that MOs in the BE range of 4–6 eV are characterized by significant contributions from Ni 3d atomic orbitals (AOs). However, the DFT calculation data showed that in this energy range there are also considerable contributions from the *2p*-AO of ligand atoms (C, O, N). This was explained by the fact that the occupied MOs in the valence band are hybridized and the valence AOs of nickel, oxygen, and nitrogen atoms are strongly mixed in them. Therefore, it is very difficult to reliably determine the energy positions of the Ni 3d-derived occupied MOs in the valence band, which is important for a complete understanding and detailed description of the electronic structure of this and other [M(Salen)] complexes, where M = 3d transition metal atom. Quantum chemical simulation methods also do not allow this to be conducted due to the significant variety of basic functions used in calculations and, as a result, the differences in the positions of the Ni 3d-derived MOs [18,19].

The main technique for obtaining information about the electronic structure of the VB is valence band photoemission spectroscopy (VB PES), which uses ultraviolet (UV) and soft X-ray photons to excite spectra [20]. It is known that the photoionization cross section of the valence AOs of different atoms varies differently with a change in the energy of exciting photons [21]. Thus, considering the energy positions of the bands in the VB PE spectrum and their behavior with a change in the energy of the exciting photons, one can obtain certain information about the origin of these bands in the VB of a polyatomic system and their relationship with the valence AOs of individual atoms. In this regard, it is advisable to excite PE spectra with photons of a wide energy range, which covers ultraviolet and soft X-ray radiation. However, even in this case, the information that is obtained about the electronic structure of the valence band from the VB PE spectra is still very limited. Additional information about the electronic structure of the valence band can be obtained using the method of resonant photoelectron spectroscopy (ResPES), which is especially useful in the study of compounds of transition and rare earth metals—particularly, 3d metals [22,23,24]. This method is implemented by measuring the VB PE spectra at photon energies in the vicinity of absorption resonances in the NEXAFS spectrum of an atom of the polyatomic system under study. The latter was previously used to investigate the electronic structure of phthalocyanines complexes of 3d atoms (Ni, Co, Fe), MPc, with a square-planar structure close to that of [Ni(Salen)] [25]. As a result, drastic changes were found in the VB PE spectra excited by photons with energies in the vicinity of *2p_3/2_ →* 3d absorption resonances in the M 2p_3/2_ NEXAFS spectra of 3d atoms, namely, a strong increase in the intensity of some PE bands in the region of low BE and the appearance of additional very intense bands at large BE. The increase in intensity is due to the superposition of two processes: (i) the direct photoionization of the occupied 3d-derived MOs and (ii) the participator-Auger decay of a resonant M *2p*_3/2_^−1^*3d*^*n*+1^ excitation, the final state of which coincides with that of the direct photoionization. In turn, additional PE bands in the VB PE spectra are due to the spectator-Auger decay of resonant excitations. Obviously, such experiments can also be informative for the [Ni(Salen)] complex as well, and can be later used to analyze the electronic structure of [M(Salen)] complexes with other 3d atoms.

This work is devoted to a detailed study of the electronic structure of the valence band of the monomeric [Ni(Salen)] complex and the role of the Ni *3d* electrons in its formation using a combination of photoemission methods. In this respect, it is a development of the photoemission study of this valence band that was carried out in [14] using only two close energies of exciting photons (75 and 150 eV), which severely limits the reliability of the conclusions drawn. To obtain more reliable information, radiation with photon energies in a wide range from 21.2 to 848 eV is exited. An analysis of the behavior of the main bands in the VB PE spectra of the [Ni(Salen)] complex with a change in the energy of the exciting photons makes it possible to determine the energy positions of the Mos that are associated with the valence C *2p* and Ni 3d states. This finding is also confirmed by a direct comparison of the VB PE spectra of the [Ni(Salen)] complex and the metal-free salen H_2_Salen (reference compound). Finally, a detailed examination of the resonant VB PE spectra excited by photons with the energies of the excited states (resonances) in the Ni 2p_3/2_, O 1s, and N 1s absorption spectra clearly indicates that the electron states at a binding energy of 3.8 eV in the VB of the [Ni(Salen)] are associated with Ni 3d-derived MOs of the complex.

## 2. Results and Discussion

**Figure 2:**
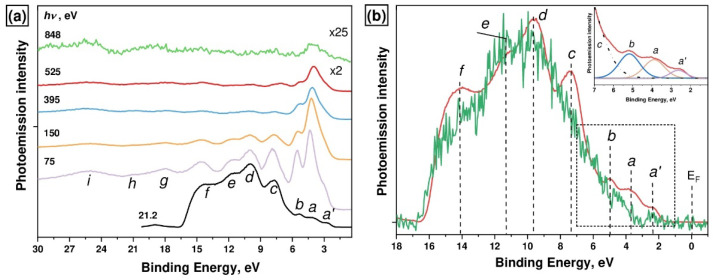
(**a**) VB PE spectra of the [Ni(Salen)] complex excited by photon energies *hν* from 21.2 eV to 848 eV; (**b**) UV PE spectra of the [Ni(Salen)] complex (red line) and the reference H_2_Salen molecule (green line) measured with *hν* = 21.2 eV. The inset shows the UV PE spectrum of the [Ni(Salen)] in the BE range from 1 to 7 eV with the fitting of components *a’*, *a*, and *b*.

The VB PE spectra of the [Ni(Salen)] complex that were measured using photons of UV (*hν* = 21.2 eV) and soft X-ray (*hν* = 75–848 eV) ranges are shown in Figure 2. As can be seen from Figure 2, all the spectra are characterized by the *a’–i* bands, except for the UV PE spectrum for which the binding energy interval is somewhat smaller, due to the low value of the exciting photon energy. These bands are the result of the photoionization of the valence hybridized MOs of the complex, which are formed by the 3d and *4s* AOs of the complexing Ni atom, as well as the *2p* and *2s* AOs of the ligand atoms (O, N, and C) [14]. It can be seen that the energy positions of the PE bands in the VB spectra remain unchanged as the energy of the exciting photons increases, while their absolute intensities decrease monotonically with an increase in photon energies *hv* from 75 to 848 eV. Moreover, the relative intensities of the PE bands change. At the same time, the PE *a’-b* bands, in contrast to the *c-f* bands, demonstrate a strong increase in intensity with an increase in the energy of the exciting photons from 21.2 eV to 75 eV. All these changes are explained by the spectral behavior of the photoionization cross-sections of the Ni 3d, O *2p,s*, N *2p,s*, and C *2p,s* subshells [21], whose electrons form the occupied valence MOs of the [Ni(Salen)] complex. Thus, in the range of *hν* from 21.2 to 848 eV, the intensity of the *a* and *b* bands first increases, reaching a maximum at *hν* = 75 eV, and then decreases monotonically. According to the data [21], it is this spectral behavior that is characteristic of the photoionization cross section of the Ni 3d subshell. Therefore, we can conclude that the low-energy *a* and *b* bands are apparently associated with MOs with dominant contributions from the Ni 3d states.

To obtain additional information about the energy position of Ni 3d-derived states, we compared the UV PE spectra for the [Ni(Salen)] complex and the metal-free salen H_2_Salen. It can be seen (Figure 2b) that both spectra are similar to each other in the BE range of 8–17 eV with PE *d-f* bands, while the main differences between them are observed for the energy range of 2–8 eV where the PE *a’–c* bands are located. Taking into account that the metal-free H_2_Salen contains 16 carbon atoms and only two oxygen and two nitrogen atoms, it is clear that the *d–f* bands are associated with the occupied σ-MOs, to which C *2s* and C *2p* AOs contribute the most. This conclusion is consistent with the results of studies [26,27,28].

The low-energy *a’*, *a*, *b*, and *c* bands are located in the UV PE spectrum of [Ni(Salen)] at the BE of 2.6, 3.8, 5.2, and 7.6 eV, respectively (see Figure 2b and inset). It should be noted that this peak fitting is somewhat different in terms of peak energy positions from that previously proposed [14]. This low-BE region is characterized by a noticeably less intense and almost structureless energy distribution of the PE signal in the H_2_Salen spectrum. In this case, comparison with the spectrum of [Ni(Salen)] is further complicated by the low quality of the metal-free salen spectrum, which is associated with sample charging during measurements. Apparently, one can only assume that the weak PE band *a’* remains, which is poorly visible in the very noisy VB PE spectrum of H_2_Salen. This PE band in the spectrum of [Ni(Salen)] was associated earlier with the photoionization of the HOMO of the complex, which originates from the π*-MOs of its phenyl groups [14].

**Figure 3:**
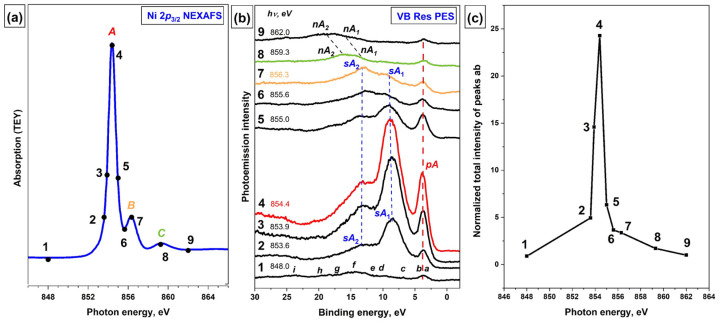
(**a**) Ni 2p_3/2_ NEXAFS spectrum, the photon energies used to excite the VB PE spectra are marked with numbered dots; (**b**) a series of resonant VB PE spectra of [Ni(Salen)]; and (**c**) the total intensity of *ab* band depending on the energy of exciting photons in the vicinity of the Ni 2p_3/2_ edge (*hν* = 848–862 eV) of the [Ni(Salen)] complex. The total intensity of the PE band *ab* at different photon energies was normalized to its intensity at *hν* = 848 eV (non-resonant excitation). *pA*—electron band due to participator-Auger decay; *sA*_1_ and *sA*_2_—low- and high-energy electron bands due to spectator Auger decay; *nA*_1_ and *nA*_2_—electron bands due to normal Auger decay of Ni *2p*_3/2_^−1^*3d*^9^ excitations.

To obtain additional information about the atomic-orbital composition of the MOs that determine the valence band of the [Ni(Salen)] complex, its resonance VB PE spectra were recorded using photons with energies in the vicinity of the main absorption resonances in the Ni 2p_3/2_, O 1s, and N 1s NEXAFS spectra of the complex. The Ni 2p_3/2_ absorption spectrum of the [Ni(Salen)] complex is shown in Figure 3a, where the photon energies that were used to excite the VB PE spectra are marked with numbered dots. The absorption resonance *A* at a photon energy of 854.4 eV (point 4) is the result of dipole-allowed transitions of the Ni 2p_3/2_ electrons to the unoccupied 3d-derived MO of *σ*-type [14]. This MO describes the σ-bonding between the complexing Ni atom and the oxygen and nitrogen atoms of the salen ligand and provides a planar-square structure of the NiN_2_O_2_ coordination center.

The next, less intense absorption band *B* at *hν* = 856.3 eV (point 7) is attributed to Ni 2p_3/2_ electron transitions to empty π*-MO, which has a strong hybridized character with the contributions from valence Ni *3d*, O *2p*, and N *2p* AOs, and reflects the presence of a π-bonding between the Ni atom and the ligand atoms (nitrogen and oxygen) in the NiN_2_O_2_ coordination center [14].

A series of VB PE spectra of [Ni(Salen)] that were excited by photons of different energies in the vicinity of the Ni 2p_3/2_ absorption edge is represented in Figure 3b. The photoemission intensities in the spectra are normalized to the incident photon flux for the convenience of their comparison. The spectra are plotted using the binding energy scale (relative to the Fermi level). Note that in this plot the PE structures stay at the same positions as the excitation photon energy changes, while the Auger peaks are linearly shifted as a function of photon energy. The bottom curve (1) in this series corresponds to *hν* = 848 eV below the Ni 2p_3/2_ absorption resonance *A*. The PE bands *a–i* in this spectrum are well consistent in their relative energy positions with those in the VB PE spectra of [Ni(Salen)] that were obtained using other photon energies (Figure 2a). It should be emphasized that PE bands *a* and *b* are not energetically resolved due to the insufficient total energy resolution of the monochromator and electron analyzer and therefore are observed in the form of one band *ab*.

As can be seen, upon excitation of the VB PE spectra by photons with energies of 853.6–855.0 eV, drastic changes are observed, namely: (i) the intensity of the PE *ab* band is significantly enhanced when the photon energy is scanned across the resonance *A*; (ii) simultaneously, the intense *sA*_1_ and *sA*_2_ bands appear additionally in the VB PE spectrum at the BE above 5 eV. For what follows, it is important to note that the *ab* band, denoted as *pA* in the ResPE spectra, retains its energy position in all spectra excited by photons of different energies, while the *sA*_1_ and *sA*_2_ bands are shifted on the BE scale according to the change in the energy of the exciting photons. The maximum enhancement of the intensity of the *ab* band reaches a value of 24 (Figure 3c) when using photons with an energy of 854.4 eV (Figure 3b, red curve 4), which corresponds to the energy of the resonance *A* in the Ni 2p_3/2_ NEXAFS spectrum of [Ni(Salen)]. After the photon energy passes resonance *A*, the intensities of the *pA*, *sA*_1_ and *sA*_2_ bands gradually decrease, and at photon energies above 859 eV (Figure 3b, curves 8 and 9), the VB PE spectrum returns to its original form if the normal Auger bands *nA*_1_ and *nA*_2_ are not considered. It should be noted that when the photon energy passes through the absorption band *B* (Figure 3b, curve 7), no additional enhancement is observed for the intensity of the PE band *ab*.

**Figure 4:**
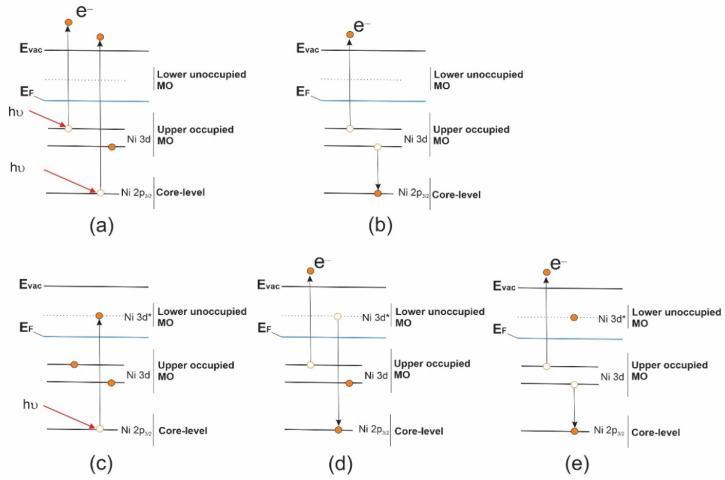
Schematic representation for photoionization absorption process (**a**), normal Auger (*nA*) electron decay process (**b**), resonant core electron excitation process (**c**), Auger electron decay processes of a resonant excitation: (**d**) participator Auger (*pA*) electron decay and (**e**) spectator Auger (*sA*) electron decay.

The appearance of new structures *pA* and *sA*_1_, *sA*_2_ in the VB PE spectra during their resonant excitation is because the absorption resonance *A* is associated with a strongly localized and long-lived core excitation *2p*_3/2_^−1^*3d*^9^ of the Ni^+2^ cation (with valence 3d^8^ electron configuration) which is formed in the process of X-ray absorption (*2p*_3/2_*3d*^8^ + *hν → 2p*_3/2_^−1^*3d*^9^). This X-ray excitation can decay through two Auger-like processes: with or without the participation of the excited Ni 2p_3/2_ electron. The first decay channel (*pA*) is the participator Auger-decay process (*2p*_3/2_^−1^*3d*^9^ → *2p*_3/2_*3d*^7^ *+ e*), whose final state coincides with that of the direct photoemission channel (*2p*_3/2_*3d*^7^ + *e*) (see the scheme of this process in Figure 4). The probability of this *pA* process is high in comparison with that of a *nA* process, since in this case the excited and Auger electrons are in the same Ni 3d electron subshell. As a result, an intense participator Auger electron signal is superimposed on that of direct photoemission (PE band *ab*), leading to a significant increase in the latter. In view of the foregoing, it is logical to assume that the *ab* band at the BE of 3.8 eV in the VB PE spectrum corresponds in the valence band to occupied MOs with dominant contributions from the Ni 3d AOs.

The second decay channel of the Ni *2p*_3/2_^−1^*3d*^9^ excitation (*sA*_1_ and *sA*_2_ bands) is a spectator Auger decay process, in which the initially excited Ni *2p_3/2_* electron does not participate but acts only as a spectator (Figure 4e). It is important to note that, these *sA*_1_ and *sA*_2_ spectator Auger bands are, in fact, bands of the normal Auger process, which are shifted in their energy positions on the BE scale to the low-energy side due to the additional screening of the Ni *2p_3/2_^−1^* vacancy by the excited electron (spectator) [29]. After the photon energy passes resonance *A*, the intensities of the *sA*_1_ and *sA*_2_ bands gradually decrease, and at photon energies above 859 eV (Figure 3b, curves 8 and 9), they pass into the normal Auger bands *nA*_1_ and *nA*_2_ (Figure 3b).

**Figure 5:**
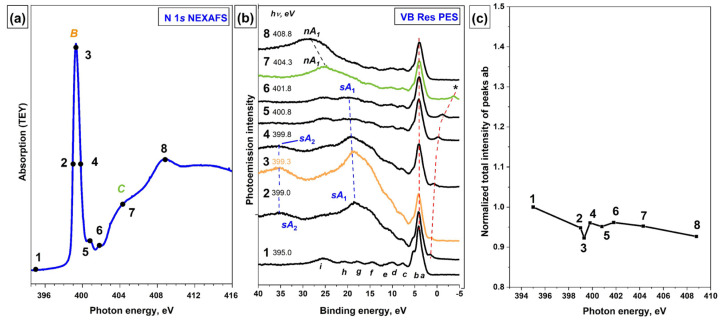
(**a**) N 1s NEXAFS spectrum, the photon energies used to excite the VB PE spectra are marked with numbered dots; (**b**) a series of resonant VB PE spectra of the [Ni(Salen)] complex and (**c**) the total intensity of the *a-b* bands depending on the energy of exciting photons in the vicinity of the N 1s absorption edge (*hν* = 395–408.8 eV) of the [Ni(Salen)] complex. In (**b**), the N 1s PE line resulting from the absorption of synchrotron radiation reflected by the diffraction grating in the second order is marked with an asterisk (*). The total intensity of the PE bands *a-b* at different photon energies was normalized to their intensity at *hν* = 395 eV (non-resonant excitation).

The N 1s NEXAFS spectrum of the [Ni(Salen)] complex is presented in Figure 5a, where the photon energies used to excite the VB PE spectra are marked with numbered dots. The intense absorption resonance *B* at *hν* = 399.3 eV is attributed to N 1s electron transitions to an empty π*-MO, which has a strong hybridized character with the contributions from valence Ni *3d*, O *2p*, and N *2p* AOs, and reflects the presence of a *π*-bonding between the Ni atom and the ligand atoms (nitrogen and oxygen) in the NiN_2_O_2_ coordination center [14]. Due to the small energy width of resonance *B*, it is obvious that the core excitation associated with it is quite long-lived. Considering this fact and the combined AO composition of this π*-MO, it is interesting to find out by what Auger processes this excitation decays.

Figure 5b presents a series of VB PE spectra for the [Ni(Salen)] complex which are measured at photon energies ranging from 395.0 to 408.8 eV. The photon energies used to excite the VB PE spectra are marked with numbered dots. It is clearly seen that as the photon energy increases, the initial VB PE spectrum (curve 1, *hν* = 399.0 eV) changes significantly in its shape due to the appearance of intense *sA*_1_ and *sA*_2_ bands (curves 2–6). The intensity of these bands reaches its maximum value at a photon energy of 399.3 eV (curve 3), at which the absorption resonance *B* occurs, and then gradually decreases (curves 4–6), after which these bands disappear and the normal Auger band *nA*_1_ appears (curves 7 and 8). It is also important that these bands are shifted in the series of the VB PE spectra towards higher binding energies that correspond to the increase in the energy of exciting photons. These findings unequivocally indicate that the *sA*_1_ and *sA*_2_ bands in the resonant VB PE spectra (Figure 5b) are the result of the spectator-Auger decay of the N *1s*^−1^π*^1^ core excitation.

It should be noted that in the presence of an intense spectator-Auger decay channel for the N *1s*^−1^π*^1^ core excitation, there is no evidence of the existence of a participator-Auger decay channel for this excitation, namely, the enhancement of the PE bands *a-b* (Figure 5c) or other PE signals in the VB PE spectra is not visible when scanning the photon energy through the absorption resonance *B*. Small variations in the intensity of bands *a-b* in the spectra excited by photons with energies in the region of resonance *B* are within the accuracy of the experiment and subsequent processing of the spectra (Figure 5c), while the overestimated intensity of these bands at *hν* = 395 eV (curve 1) is the result of the superposition of the core N 1s PE line on this band. The latter is a result of the synchrotron radiation absorption, which is reflected by the monochromator diffraction grating in the second order of diffraction.

**Figure 6:**
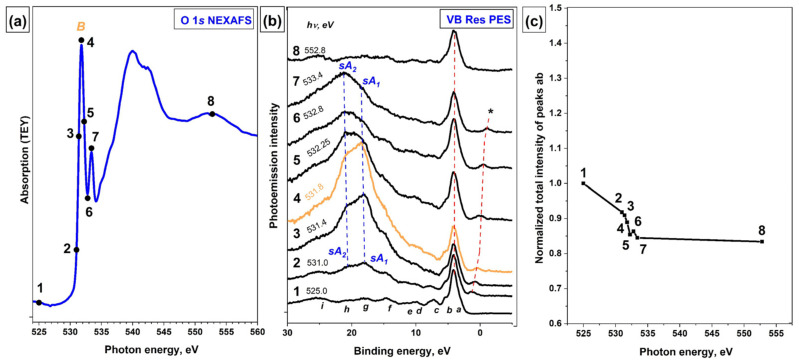
(**a**) O 1s NEXAFS spectrum, the photon energies *hν* used to excite the VB PE spectra are marked with numbered dots; (**b**) a series of resonant VB PE spectra of the [Ni(Salen)] complex; and (**c**) the total intensity of the *a-b* bands depending on the energy of exciting photons in the vicinity of the O 1s absorption edge (*hν* = 525–552.8 eV) of the [Ni(Salen)] complex. In (**b**), the O 1s PE line resulting from the absorption of synchrotron radiation reflected by the diffraction grating in the second order is marked with an asterisk (*). The total intensity of the PE bands *a-b* at different photon energies was normalized to their intensity at *hν* = 525 eV (non-resonant excitation).

Finally, let us consider the VB PE spectra of the [Ni(Salen)] complex, which were recorded using the photon energies *hν* in the vicinity of the *B* resonance in the O 1s NEXAFS spectrum of this complex (Figure 6a). In Figure 6, the photon energies used to excite the VB PE spectra are marked with numbered dots. As in the case of N 1s NEXAFS spectrum (Figure 5a), the O 1s absorption spectrum is also dominated by an intense resonance *B* that is located at a photon energy of 531.8 eV. This resonance is similar in nature to the *B* resonances in the Ni 2p_3/2_ and N 1s NEXAFS spectra and is associated with electron transitions from the O 1s core level to the lower unoccupied *π*-type MO containing contributions from the valence Ni *3dπ*, O *2pπ* and N *2pπ* AOs [14].

Figure 6b shows a series of VB PE spectra that were recorded in the vicinity of the O 1s X-ray absorption edge at the photon energies of 525–552.8 eV. As in the case of the VB PE spectra that were measured using photons with energies in the vicinity of the N 1s absorption edge (Figure 5), the PE bands *a*-*b* or any other PE signals in the VB PE spectra do not show an increase in intensity when scanning the photon energy through the absorption resonance *B* (see Figure 6b,c). Small changes in the intensity of the bands *a-b* in the spectra excited by photons with energies in the vicinity of resonance *B* are within the accuracy of the experiment and subsequent processing of the spectra (Figure 6c), while the overestimated intensity of these bands at *hν* = 525 eV (curve 1) is the result of superposition of the core O 1s PE line on this band. This PE line is a result of absorption of synchrotron radiation, which is reflected by the monochromator diffraction grating in the second order of diffraction.

Thus, only additional intense bands *sA*_1_ and *sA*_2_ are observed in the VB PE spectra of the [Ni(Salen)] complex when they are excited by photons in the vicinity of the O 1s absorption edge. They appear in the spectrum at *hν* = 531 eV (curve 2), grow in intensity (curves 2–4) up to an energy of 531.8 eV, at which the absorption resonance *B* is located, and then decrease in intensity (curves 5–7), finally disappearing in the spectrum at *hν* = 552.8 eV (curve 8). It is important that these bands are shifted in the series of the VB PE spectra towards higher binding energies corresponding to the increase in the energy of the exciting photons. In view of the foregoing, it is obvious that the *sA*_1_ and *sA*_2_ bands in the resonant VB PE spectra (Figure 6b) are the result of the spectator-Auger decay of the O *1s*^−1^π*^1^ excitation. It should be noted that the absence of the participator-Auger decay band in the VB PE spectra is observed for the absorption resonance *B* in the Ni 2p_3/2_, N 1s, and O 1s NEXAFS spectra of the [Ni(Salen)] complex. All these resonances are associated with the transitions of the core electrons to the same π*-MO with a strongly hybridized character. Since the energy widths of these resonances in all spectra are small, they are associated with long-lived core excitations, for which both the spectator- and participator-Auger decay processes of excitation usually take place. The absence of enhancement of the PE bands *a*-*b* or other PE signals in the VB PE spectra during the Auger decay of core excitations that are responsible for the *B* band seems to be a fundamental problem that is associated with the *π*-symmetry of the *B* excitation (in contrast to the *σ*-symmetry for the *A* excitation), and this requires additional study.

## 3. Materials and Methods

The metal-free H_2_Salen (N,N’-bis(salicylidene)-ethylene-1,2-diamine) was synthesized according to the procedures [30,31,32] by condensation of salicylaldehyde and 1,2-ethylenediamine in EtOH under reflux for 3 h. After reaching room temperature, the mixture was cooled in the freezer. The insoluble precipitate was then filtered off and recrystallized from EtOH to give a yellow polycrystalline solid. The [Ni(Salen)] molecular complexes were synthesized in a powder form using the standard procedure by adding nickel(II) acetate tetrahydrate to the ligand (H_2_Salen) in ethanol [32,33]. The obtained powder was purified via recrystallization from EtOH, followed by drying under vacuum at 80 °C.

The UV PE study of the valence band for the [Ni(Salen)] complex and the H_2_Salen reference were carried out with the He(I) resonance radiation (*hν* = 21.2 eV) using the Thermo Fisher Scientific ESCALAB 250 Xi laboratory spectrometer (at the Resource center “Physical methods of surface investigation”, St. Petersburg State University). The soft X-ray VB PE and the ResPE spectra from [Ni(Salen)], along with auxiliary NEXAFS spectra, were measured using monochromatic synchrotron radiation (SR) and facilities of Russian-German beamline (RGBL) at the BESSY II electron storage ring, (Berlin, Germany) [34].

The [Ni(Salen)] and H_2_Salen samples were prepared in situ by thermal evaporation of a thoroughly dehydrated powder from a tantalum or quartz crucible and deposition onto clean polycrystalline Pt or Ti plates under ultrahigh vacuum conditions (about 10^–9^ mbar). The heating temperature of the crucible was about 550 K; the layer thickness was about 5–10 nm. According to mass spectrometry data, the vapor phase over solid [Ni(salen)] is composed of monomer molecules, i.e., sublimation is preceded by destruction of dimer fragments in crystal [35]. The purity and chemical composition of the prepared samples were controlled by measuring survey and core PE spectra using the monochromatic Al Kα radiation (ESCALAB 250 Xi) and SR (RGBL). These spectra for samples are given in the Supplementary Materials (see Figures S1–S3).

All VB PE spectra were measured in the normal photoemission geometry and the angle-integrated mode using a double-focusing full 180° spherical sector electron analyzer (ESCALAB 250 Xi) and a PHOIBOS 150 hemispherical electron analyzer (Specs GmbH, Berlin, Germany). The VB PE spectra that were obtained using a laboratory spectrometer were measured in the Constant Analyzer Energy (CAE) mode at a pass energy of 1 eV. The total energy resolution was ~360 meV. When recording VB PE spectra using PHOIBOS 150, the analyzer operated in the CAE mode, and the pass energy was 10 eV. Then, the obtained spectra were normalized to the intensity of the incident photon flux. The monochromator energy resolution was 15–340 meV using excitation radiation with a photon energy of 75–862 eV, respectively.

The NEXAFS spectra that were required for ResPES measurements were recorded in the total electron yield mode by measuring the sample drain current at a synchrotron beam incidence angle of 45°. The monochromator resolution in the vicinity of Ni 2p_3/2_ (~850 eV); O 1s (~530 eV); and N 1s (~400 eV) was 300, 150, and 90 meV, respectively. The photon energy in the range of 70–950 eV was calibrated by measuring the Pt 4f_7/2_ PE spectra excited by the radiation which was reflected by a diffraction grating in the first and second diffraction orders. All ResPES and NEXAFS measurements were performed under UHV conditions (~2·10^−10^ mbar) at room temperature.

## 4. Conclusions

A combination of ultraviolet, soft X-ray, and resonant photoemission methods was used to study the electronic structure of the valence band of the [Ni(Salen)] complex. For this, the VB PE spectra of the latter were recorded using He(I) resonance and synchrotron radiation with photon energies ranging from 21.2 eV to 860 eV. A comparative analysis of the VB PE spectra of metal-free H_2_Salen and [Ni(Salen)] revealed that the PE bands observed in the binding energy range of 8–17 eV are mainly associated with *σ*-MOs in which the contributions from the C *2s* and C *2p* AOs dominate. In turn, the low energy range of 2–6 eV contains bands that are associated with MOs with dominant contributions from the Ni 3d AOs. Information about the atomic–orbital composition of MOs that determine the VB of the [Ni(Salen)] complex was obtained by analyzing its VB PE resonance spectra using photons with energies in the vicinity of the main absorption resonances in the Ni 2p_3/2_, O 1s, and N 1s NEXAFS spectra. New electronic bands were found in the VB PE spectra of [Ni(Salen)] that were measured using photons with resonance *A* energy (854.4 eV) in the Ni 2p_3/2_ NEXAFS spectrum. These bands have been attributed to the participator- and spectator-Auger decay channels of the core Ni *2p*_3/2_^−1^*3d*^9^ excitation that are responsible for the absorption resonance *B*. The participator-Auger decay channel manifests itself in the VB PE spectra as a significant increase in the intensity of the PE band *ab*, which is considered as confirmation of the dominant 3d composition of occupied MOs, the direct photoionization of which leads to the appearance of the PE band *ab* at the BE of 3.8 eV. In the VB PE spectra, which were excited by photons with energies of 399.4 (N 1s NEXAFS); 531.8 (O 1s NEXAFS); and 856.3 eV (Ni 2p_3/2_ NEXAFS) corresponding to resonance *B*, there are only spectator-Auger decay channels. The absence of enhancement of the PE bands *a*-*b* or other PE signals in the VB PE spectra during the Auger decay of core excitations that are responsible for the *B* band seems to be a fundamental problem that is associated with the *π*-symmetry of the *B* excitation (in contrast to the *σ*-symmetry for the *A* excitation), and it requires additional study.

## Data Availability

Not applicable.

## Supplementary Materials

The following supporting information can be downloaded at: https://www.mdpi.com/article/10.3390/ijms23116207/s1.
