# Supplementary material for: The Valence Band Structure of the [Ni(Salen)] Complex: An Ultraviolet, Soft X-ray and Resonant Photoemission Spectroscopy Study
Source: Int J Mol Sci. 2022 Jun 1;23(11):6207. doi: 10.3390/ijms23116207 (PMC9181502; doi:10.3390/ijms23116207)
Supplement: Supplementary file 1 [file ijms-23-06207-s001.zip › ijms-1722116-supplementary.pdf]

International Journal of *Molecular Sciences*

MDPI

Supplementary information

Article

The valence band structure of the [Ni(Salen)] complex: an ultra-violet, soft X-ray and resonant photoemission spectroscopy study

Petr M. Korusenko [1,2*], Alexandra V. Koroleva [3], Anatoliy A. Vereshchagin [4], Danil V. Sivkov [1,5], Olga V. Petrova [1,5], Oleg V. Levin [4], and Alexander S. Vinogradov [1]

[1] Department of Solid State Electronics, St. Petersburg State University, 7/9 Universitetskaya nab., 199034 Saint Petersburg, Russia
[2] Department of Physics, Omsk State Technical University, 11 Mira prosp., 644050 Omsk, Russia
[3] Resource center "Physical methods of surface investigation", St. Petersburg State University, St. Petersburg State University, 7/9 Universitetskaya nab., 199034 Saint Petersburg, Russia
[4] Department of Electrochemistry, Institute of Chemistry, St. Petersburg State University, 7/9 Universitetskaya nab., 199034 Saint Petersburg, Russia
[5] Institute of Physics and Mathematics, Komi Science Centre of the Ural Branch of the Russian Academy of Sciences, 167982 Syktyvkar, Russia
* Correspondence: p.korusenko@spbu.ru (P.M. Korusenko)

As can be seen from Figure S1, all presented survey spectra of H_2Salen and the [Ni(Salen)] complex do not show any peaks from substrate elements or contaminant inclusions. Therefore, it can be stated that the monomer layers thus prepared are of high purity.

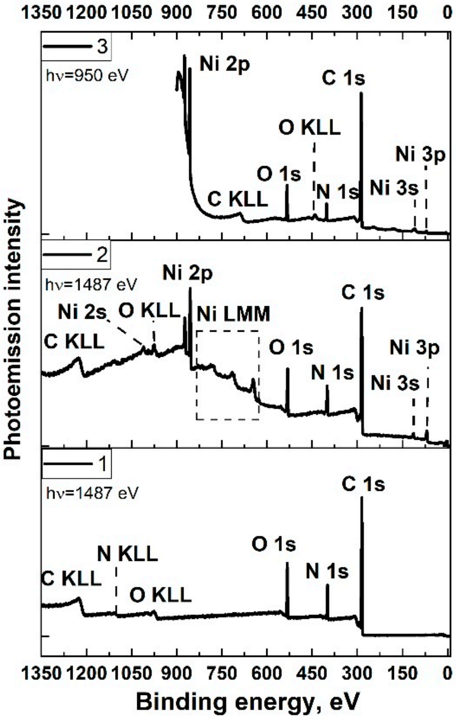

Figure S1. Survey PE spectra from the metal-free H_2Salen (1) and [Ni(Salen)] complex deposited onto different substrate: (2) – Ti foil (ESCALAB 250 Xi); (3) – Pt plate (BESSY II electron storage ring, Russian-German beamline (RGBL))

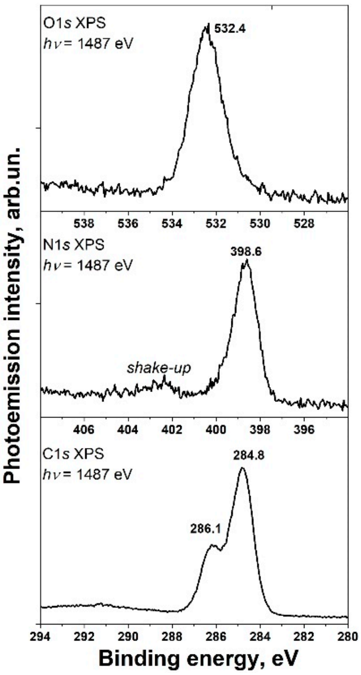

Figure S2. O 1s, N 1s and C 1s PE spectra of the H_2Salen deposited onto Ti foil (ESCALAB 250 Xi)

It can be seen from Fig. S3 that the Ni $2p_{3/2;1/2}$, O 1s, N 1s and C 1s PE spectra measured from [Ni(Salen)] deposited onto the Pt and Ti substrates practically do not differ in shape, but differ in the position of the peak maxima. This shift is |0.3| eV and is the result of differences in the calibration of the spectrometers.

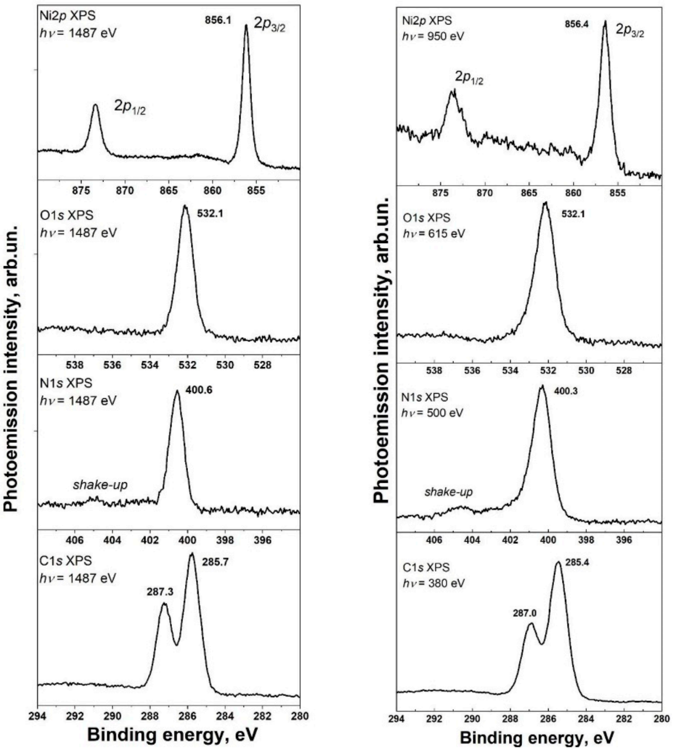

(a) (b)

Figure S3. Ni $2p_{3/2;1/2}$, O 1s, N 1s and C 1s PE spectra of the [Ni(Salen)] complex deposited onto different substrate: (a) – – Ti foil (ESCALAB 250 Xi); (b) –Pt plate (BESSY II electron storage ring, Russian-German beamline (RGBL))
